# Association Between the Triglyceride-to-HDL Cholesterol Ratio and Glycemic Control in Patients with Type 2 Diabetes Mellitus: A Real-World Retrospective Study

**DOI:** 10.3390/biomedicines14091995

**Published:** 2026-09-04

**Authors:** Ali Erol, Muhammet Fatih Şahin

**Affiliations:** 1 Department of Internal Medicine, Bursa Yüksek İhtisas Training and Research Hospital, 16100 Bursa, Türkiye; 2 Department of Internal Medicine, Kestel State Hospital, 16100 Bursa, Türkiye

**Keywords:** dyslipidemia, glycemic control, HbA1c, real-world evidence, triglyceride-to-HDL cholesterol ratio, type 2 diabetes mellitus

## Abstract

**Background and Objectives**: The triglyceride-to-high-density lipoprotein cholesterol ratio (TG/HDL-C) is a simple lipid index associated with insulin resistance and atherogenic dyslipidemia. Previous evidence has supported the TG/HDL-C ratio as a simple surrogate marker of insulin resistance and an adverse metabolic profile. However, its relationship with glycemic control in real-world patients with type 2 diabetes mellitus (T2DM) remains uncertain. This study evaluated the association between the TG/HDL-C ratio and poor glycemic control in patients with T2DM. **Materials and Methods**: This retrospective observational study included patients with T2DM followed in outpatient clinics. Poor glycemic control was defined as glycated hemoglobin A1c (HbA1c) ≥ 7%. The association between the TG/HDL-C ratio and glycemic control was assessed using group comparisons, Spearman correlation, tertile analysis, and multivariable logistic regression adjusted for age, sex, LDL-C, total cholesterol, insulin use, statin use, and fenofibrate use. Receiver operating characteristic (ROC) analysis was used to evaluate the discriminatory performance of the TG/HDL-C ratio for identifying poor glycemic control. A sensitivity analysis was performed after excluding patients receiving fenofibrate. **Results**: A total of 1001 patients with T2DM were included, of whom 568 (56.7%) had poor glycemic control. The mean age was 60.9 ± 11.4 years, and 57.1% of participants were women. The poor- and adequate-glycemic-control groups were comparable with respect to age and sex. The TG/HDL-C ratio was higher in patients with HbA1c ≥7% than in those with HbA1c < 7% [3.35 (2.35–5.00) vs. 2.74 (1.87–3.82), *p* < 0.001] and increased progressively across tertiles. In multivariable analysis, the TG/HDL-C ratio remained associated with poor glycemic control after adjustment for available covariates (OR: 1.201, 95% CI: 1.125–1.282, *p* < 0.001). The association remained significant after excluding fenofibrate users. ROC analysis showed modest discrimination (AUC: 0.601, 95% CI: 0.566–0.636). **Conclusions**: The TG/HDL-C ratio was associated with poor glycemic control in patients with T2DM, showing a graded association across tertiles. However, its standalone discriminatory performance is modest. Given its modest discriminatory performance, the TG/HDL-C ratio should not be considered a standalone diagnostic or predictive tool for poor glycemic control; rather, it may serve as an accessible adjunctive metabolic marker associated with an adverse glycemic and lipid profile.

## 1. Introduction

Type 2 diabetes mellitus (T2DM) is one of the most important chronic metabolic diseases worldwide and is associated with a substantial burden of microvascular and macrovascular complications [1,2]. According to the 11th edition of the International Diabetes Federation Diabetes Atlas, approximately 589 million adults aged 20–79 years were living with diabetes worldwide in 2024, corresponding to 11.1% of the adult population, and more than 90% of diabetes cases are attributable to T2DM [3]. In addition to chronic hyperglycemia, patients with T2DM frequently exhibit a cluster of metabolic abnormalities, including insulin resistance, dyslipidemia, central adiposity, and chronic low-grade inflammation. Recent real-world evidence has also demonstrated a substantial burden of both microvascular and macrovascular complications among individuals with T2DM, further emphasizing the importance of identifying readily available metabolic markers associated with adverse metabolic profiles. [4] Among these abnormalities, diabetic dyslipidemia is particularly important because it contributes directly to cardiovascular morbidity and mortality, which remain leading causes of adverse outcomes in this population [5,6,7].

Diabetic dyslipidemia is typically characterized by elevated triglyceride levels, reduced high-density lipoprotein cholesterol (HDL-C) levels, and qualitative changes in low-density lipoprotein cholesterol (LDL-C) particles [7,8,9,10]. Although LDL-C remains the primary lipid target in most cardiovascular prevention strategies, triglyceride-rich lipoproteins and low HDL-C also reflect an atherogenic and insulin-resistant metabolic state. Therefore, lipid indices that integrate different components of diabetic dyslipidemia may provide additional information beyond individual lipid parameters alone [11,12].

The triglyceride-to-HDL cholesterol (TG/HDL-C) ratio is a simple and easily calculated index derived from routine lipid measurements. It has been proposed as a surrogate marker of insulin resistance, atherogenic dyslipidemia, metabolic syndrome, and cardiometabolic risk [13,14,15,16]. Meta-analytic evidence from broader populations suggests that an elevated TG/HDL-C ratio is associated with an increased risk of cardiovascular events [17]. However, large-scale meta-analytic evidence specifically evaluating this association in patients with T2DM remains comparatively limited, highlighting the need for further studies in well-characterized diabetic populations. In patients with T2DM, the TG/HDL-C ratio may be particularly relevant because both hypertriglyceridemia and low HDL-C are closely linked to impaired insulin action, hepatic lipid overproduction, and worsening metabolic control [18]. Several studies have suggested that a higher TG/HDL-C ratio is associated with insulin resistance and adverse cardiometabolic risk profiles [19,20,21,22].

Glycated hemoglobin A1c (HbA1c) is widely used to assess long-term glycemic control and to guide treatment decisions in patients with T2DM [22]. Poor glycemic control is associated with a higher risk of diabetes-related complications and often coexists with a more unfavorable lipid profile. However, the relationship between the TG/HDL-C ratio and glycemic control in real-world T2DM populations has not been fully clarified. In particular, it remains uncertain whether the TG/HDL-C ratio is associated with poor glycemic control after accounting for lipid parameters and treatment-related factors [23,24,25,26,27].

A previously published study using the same real-world T2DM cohort primarily evaluated patterns of lipid-lowering therapy, guideline concordance, and clinical factors associated with the use of statins and fibrates [28]. In contrast, the present study addresses a distinct research question and outcome framework. Specifically, the current analysis focuses on the association between the TG/HDL-C ratio and glycemic control, with poor glycemic control defined as HbA1c ≥ 7%. The principal analyses—including correlation analysis, TG/HDL-C tertile comparisons, receiver operating characteristic analysis, and multivariable logistic regression for poor glycemic control—were not the primary objectives of the previous report. Thus, although the two studies originate from the same source cohort, they address different clinical questions, outcomes, and analytical objectives.

## 2. Materials and Methods

### 2.1. Study Design and Setting

This retrospective observational study was conducted using electronic medical records of patients with type 2 diabetes mellitus (T2DM) who were followed in the Internal Medicine outpatient clinics of Kestel State Hospital and Bursa Yüksek İhtisas Training and Research Hospital, Bursa, Türkiye. The study included patients who attended routine outpatient visits between March 2024 and March 2025 and was reported in accordance with the Strengthening the Reporting of Observational Studies in Epidemiology (STROBE) statement [29]. The study protocol was approved by the Institutional Ethics Committee of Bursa Yüksek İhtisas Training and Research Hospital (approval number: 2024-TBEK 2026/06-07) and was conducted in accordance with the principles of the Declaration of Helsinki. Given the retrospective design of the study and the use of anonymized data, the requirement for written informed consent was waived. Patient confidentiality was maintained throughout all stages of data handling and analysis.

The present study was designed as a secondary analysis of an anonymized real-world T2DM dataset that was previously used in a published study evaluating lipid-lowering treatment patterns and guideline concordance in patients with T2DM [28]. The previously published study primarily focused on the use of lipid-lowering therapies, including statins and fibrates, treatment thresholds, and predictors of medication use. In contrast, the present analysis addresses a distinct research question: whether the TG/HDL-C ratio is associated with glycemic control and whether it can identify patients with poor glycemic control, defined as HbA1c ≥ 7%. Therefore, the current study does not re-evaluate guideline adherence or predictors of lipid-lowering therapy initiation, but instead investigates the TG/HDL-C ratio as a metabolic marker of glycemic status.

### 2.2. Study Population

A total of 1001 adult patients with T2DM were included. Eligible patients were adults aged 18–90 years with a confirmed diagnosis of T2DM and available laboratory data, including HbA1c, fasting plasma glucose, triglycerides, HDL-C, LDL-C, and total cholesterol. Patients with type 1 diabetes mellitus, gestational diabetes, missing essential laboratory data, or severe comorbid conditions that could substantially interfere with lipid or metabolic measurements were excluded. No additional patient contact, intervention, or new data collection was performed for the present study; all analyses were conducted using existing anonymized electronic medical records. The previous publication did not primarily investigate the TG/HDL-C ratio in relation to glycemic control and did not use HbA1c-defined poor glycemic control as the principal outcome. The present secondary analysis therefore addresses a distinct hypothesis and analytical framework focused specifically on the relationship between the TG/HDL-C ratio and glycemic status. No a priori sample-size calculation was performed because the study was retrospective and all eligible patient records meeting the inclusion criteria during the predefined study period were included. The final sample size therefore reflected the total number of eligible patients available in the source population during the study period.

### 2.3. Data Collection and Study Variables

Demographic, clinical, laboratory, and treatment-related data were retrospectively retrieved from the electronic hospital record system. The treatment-related variables available in the dataset included oral antidiabetic drug use (recorded as a binary yes/no variable without class-specific categorization), insulin use, statin use, fenofibrate use, gemfibrozil use, ezetimibe use, and medication use for diabetic neuropathy. Specific glucose-lowering drug classes, including SGLT2 inhibitors, GLP-1 receptor agonists, semaglutide, tirzepatide, and other individual antidiabetic agents, could therefore not be evaluated separately. Fasting plasma glucose and lipid parameters were obtained from blood samples collected after at least 8 h of fasting, and HbA1c values recorded during the same clinical assessment period were used for analysis. Medication exposure was defined according to documented insulin, statin, and fenofibrate use at the time of the corresponding clinical assessment. The exact dates of treatment initiation and treatment duration relative to the laboratory measurements were not available for the present analysis. The TG/HDL-C ratio was calculated for each patient as triglycerides divided by HDL-C, with both expressed in mg/dL. Poor glycemic control was defined as HbA1c ≥ 7%, whereas HbA1c < 7% was considered better glycemic control. Insulin use was recorded as the treatment status documented in the available medical records; however, the exact date of insulin initiation and treatment duration relative to the HbA1c measurement were not available for the present analysis. No patient in the cohort was receiving PCSK9 inhibitor therapy during the study period; consequently, PCSK9 inhibitor use could not be included in the statistical analyses. For the present secondary analysis, class-specific coding of individual glucose-lowering therapies was not available in a form that allowed separate evaluation of specific drug classes. The analyzable lipid-lowering treatment variables included statin, fenofibrate, gemfibrozil, and ezetimibe use.

### 2.4. Grouping According to TG/HDL-C Ratio

The TG/HDL-C ratio was analyzed primarily as a continuous variable to preserve the full information contained in the measure. In addition, patients were categorized into tertiles for descriptive and secondary analyses to assess whether glycemic and metabolic parameters exhibited a graded pattern across increasing levels of the TG/HDL-C ratio. The tertile-based analysis was therefore intended to complement, rather than replace, the primary continuous-variable analysis. Poor glycemic control was defined as HbA1c ≥ 7%, based on the general glycemic target recommended by the American Diabetes Association Standards of Care in Diabetes—2026 for many nonpregnant adults with diabetes [22].

### 2.5. Statistical Analysis

All statistical analyses were performed using IBM SPSS Statistics version 22.0. The distribution of continuous variables was assessed using the Shapiro–Wilk test together with visual inspection of histograms and Q–Q plots. Normally distributed continuous variables were presented as mean ± standard deviation, whereas non-normally distributed variables were presented as median and interquartile range (IQR), defined as the 25–75th percentiles (Q1–Q3). Categorical variables were presented as number and percentage. Comparisons between patients with HbA1c < 7% and HbA1c ≥ 7% were performed using the Mann–Whitney U test for non-normally distributed continuous variables and the chi-square test for categorical variables. Comparisons across TG/HDL-C tertiles were performed using the Kruskal–Wallis test for continuous variables and the chi-square test for categorical variables. When the overall Kruskal–Wallis test was significant, pairwise Mann–Whitney U tests were performed with Bonferroni correction for three comparisons (adjusted significance threshold, *p* < 0.0167). Pairwise effect sizes were calculated as r = |Z|/√N. Correlations between the TG/HDL-C ratio and clinical–metabolic parameters were assessed using Spearman’s rank correlation analysis.

Receiver operating characteristic (ROC) curve analysis was performed to evaluate the discriminatory ability of the TG/HDL-C ratio for identifying poor glycemic control. The area under the curve (AUC), 95% confidence interval (CI), and *p*-value were reported, and the optimal cut-off value was selected according to the Youden index [30]. Multivariable logistic regression analyses were performed to determine whether the TG/HDL-C ratio remained associated with poor glycemic control after adjustment for available covariates. The primary multivariable analysis modeled the TG/HDL-C ratio as a continuous variable. A secondary tertile-based model was additionally constructed to evaluate the presence of a graded association across increasing TG/HDL-C categories, with the lowest tertile used as the reference category. Covariates were selected a priori based on their clinical relevance and their potential to confound the association between the TG/HDL-C ratio and glycemic control, rather than solely on statistical significance in univariable analyses. Both models were adjusted for age, sex, LDL-C, total cholesterol, insulin use, statin use, and fenofibrate use, and results were expressed as odds ratios (ORs) with 95% CIs. Multicollinearity was evaluated using tolerance and variance inflation factor (VIF) values, with VIF below 3.0 considered to indicate the absence of relevant multicollinearity. Internal validation of the multivariable logistic regression model was performed using 1000 bootstrap resamples with bias-corrected and accelerated 95% confidence intervals.

Because fenofibrate therapy may directly influence triglyceride and HDL-C levels, an additional sensitivity analysis was performed after excluding patients receiving fenofibrate therapy, repeating both the continuous and tertile-based models [31]. A two-sided *p*-value of <0.05 was considered statistically significant.

## 3. Results

### 3.1. Participant Characteristics and Glycemic-Control Groups

A total of 1001 patients with T2DM were included in the final analysis. The mean age was 60.9 ± 11.4 years, and 572 patients (57.1%) were female. The median HbA1c level was 7.2 (6.3–8.3)%, and the median fasting plasma glucose level was 134 (110–169)mg/dL. The median TG/HDL-C ratio was 3.16 (interquartile range [IQR]: 2.11–4.75). Overall, 184 patients (18.4%) were receiving insulin therapy, 299 (29.9%) statin therapy, and 93 (9.3%) fenofibrate therapy. Baseline clinical and laboratory characteristics are presented in Table 1.

Poor glycemic control, defined as HbA1c ≥ 7%, was present in 568 patients (56.7%), whereas 433 patients (43.3%) had HbA1c < 7%. Patients with poor glycemic control had significantly higher fasting plasma glucose levels [158.0 (124.0–197.0) vs. 114.0 (98.0–134.0) mg/dL, *p* < 0.001]. The TG/HDL-C ratio was also significantly higher in patients with HbA1c ≥ 7% [3.35 (2.35–5.00) vs. 2.74 (1.87–3.82), *p* < 0.001]. In addition, HDL-C levels were lower, whereas triglyceride levels were higher, in the poor glycemic control group. Insulin use was more frequent among patients with HbA1c ≥ 7% (25.2% vs. 9.5%, *p* < 0.001). No significant differences were observed between the two groups in sex distribution, neuropathy drug use, fenofibrate use, or statin use. These comparisons are shown in Table 2.

### 3.2. Correlation Analysis

Spearman correlation analysis showed that the TG/HDL-C ratio was positively correlated with HbA1c (rho = 0.172, *p* < 0.001) and fasting plasma glucose (rho = 0.168, *p* < 0.001), and positively correlated with total cholesterol (rho = 0.208, *p* < 0.001). As expected, because triglycerides and HDL-C are the mathematical components of the TG/HDL-C ratio, the index was positively correlated with triglycerides (rho = 0.895, *p* < 0.001) and inversely correlated with HDL-C (rho = −0.635, *p* < 0.001); these component correlations are inherent to the construction of the index and are therefore not tabulated as primary findings in Table 3. No significant correlation was observed between the TG/HDL-C ratio and LDL-C (rho = −0.008, *p* = 0.789). Correlation results are presented in Table 3.

### 3.3. TG/HDL-C Tertile Analysis

**Figure 1:**
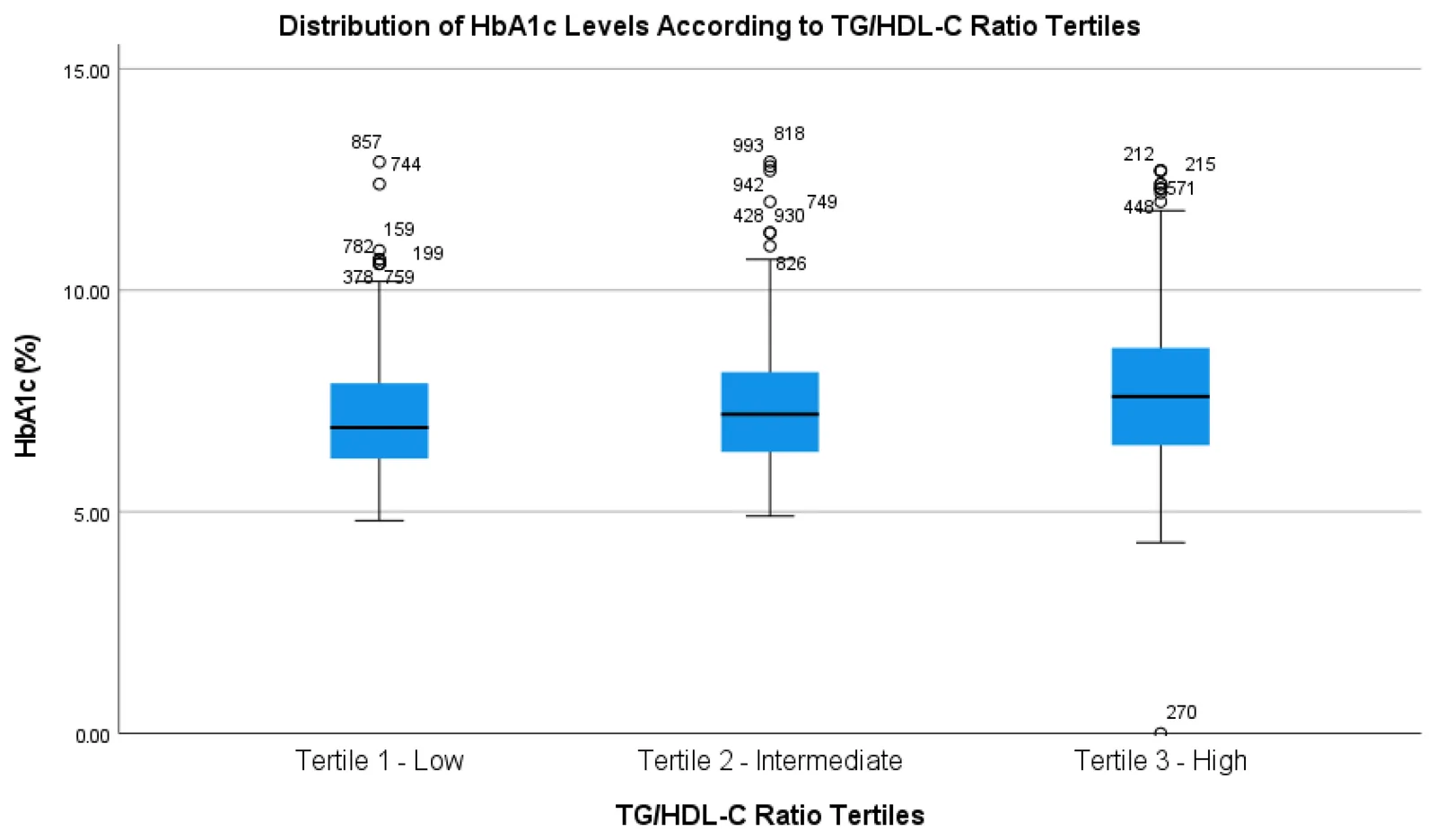
Distribution of HbA1c levels according to TG/HDL-C ratio tertiles. Notes: Boxplot showing HbA1c levels across TG/HDL-C ratio tertiles. HbA1c levels increased progressively from the lowest to the highest TG/HDL-C ratio tertile. The difference among tertiles was statistically significant according to the Kruskal–Wallis test (*p* < 0.001).

Patients were divided into three tertiles according to the TG/HDL-C ratio: low (n = 333), intermediate (n = 335), and high (n = 333). HbA1c levels increased progressively across tertiles [6.90 (6.20–7.90), 7.20 (6.30–8.20), and 7.60 (6.50–8.70), respectively; *p* < 0.001], as did fasting plasma glucose [124.0 (105.0–157.0), 138.0 (114.0–169.0), and 146.0 (113.5–192.0) mg/dL, respectively; *p* < 0.001]. As expected, triglyceride levels increased markedly across tertiles, whereas HDL-C levels decreased progressively, and total cholesterol also differed significantly (*p* < 0.001). Female sex was more frequent in the lowest tertile and decreased across tertiles (63.1%, 57.0%, and 51.4%; *p* = 0.009). Fenofibrate use was substantially more frequent in the highest tertile (1.2%, 5.7%, and 21.0%; *p* < 0.001), whereas statin use was less frequent in the highest tertile (33.3%, 34.3%, and 21.9%; *p* = 0.001). Insulin use did not differ across tertiles (*p* = 0.451). Post-hoc pairwise comparisons with Bonferroni correction showed that HbA1c was significantly higher in the third tertile than in the first (*p* < 0.001) and second tertiles (*p* = 0.001), whereas the difference between the first and second tertiles was not statistically significant (*p* = 0.051). The corresponding pairwise effect sizes were small (r = 0.20, r = 0.13, and r = 0.08, respectively). These findings are summarized in Table 4 and Figure 1.

### 3.4. ROC Analysis of the TG/HDL-C Ratio

**Figure 2:**
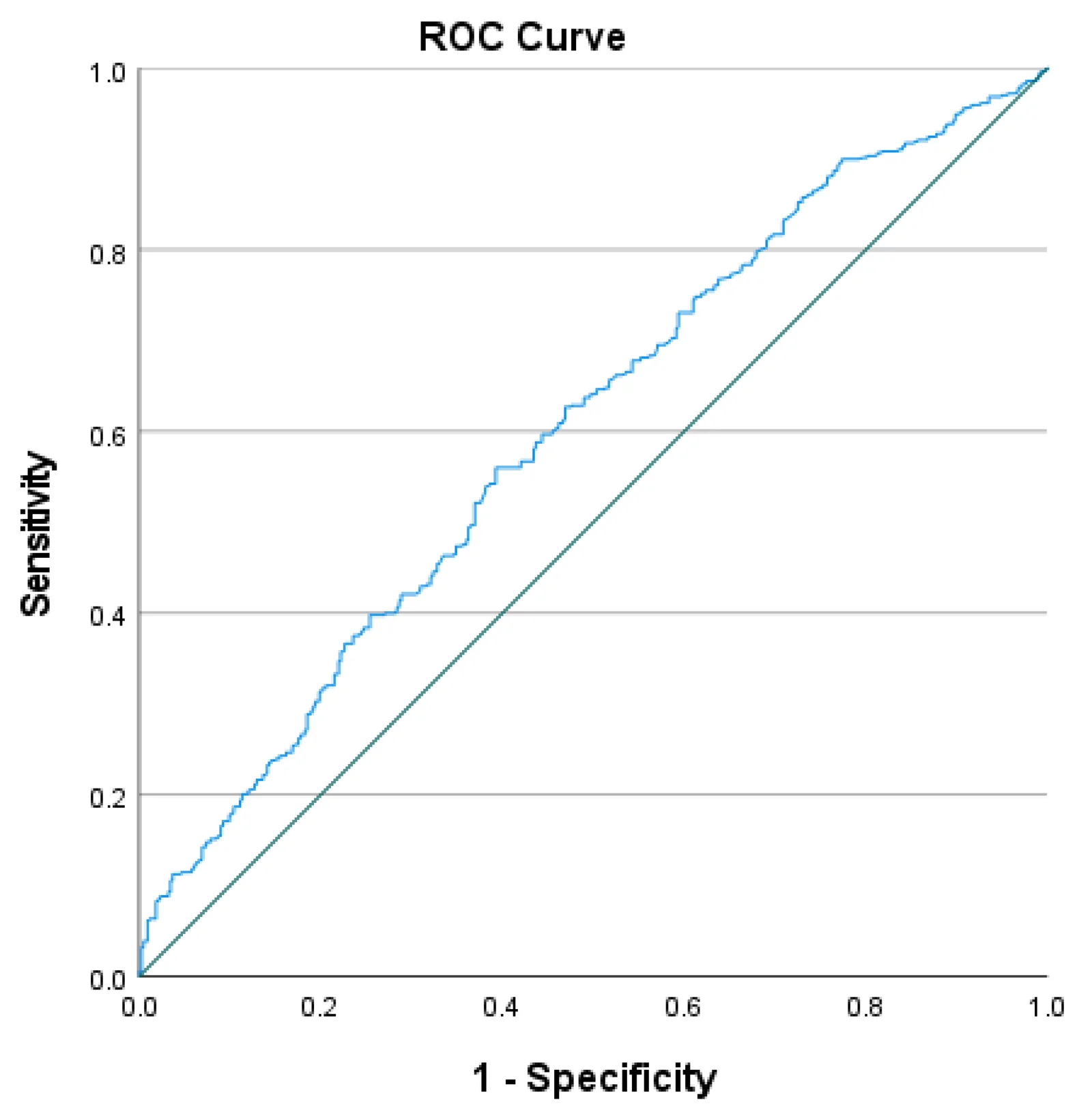
ROC curve of the TG/HDL-C ratio for discriminating poor glycemic control. Notes: Receiver operating characteristic curve of the TG/HDL-C ratio for identifying poor glycemic control defined as HbA1c ≥ 7%. The TG/HDL-C ratio showed a statistically significant but modest discriminatory ability, with an AUC of 0.601 (95% CI: 0.566–0.636, *p* < 0.001). The optimal cut-off value was 3.06, with a sensitivity of 58.1% and specificity of 56.6%.

ROC analysis showed statistically significant but limited discrimination of HbA1c ≥ 7% by the TG/HDL-C ratio (AUC: 0.601, 95% CI: 0.566–0.636, *p* < 0.001). At the Youden-derived cut-off of 3.06, sensitivity was 58.1% and specificity was 56.6%, indicating limited standalone classification performance. The ROC curve is shown in Figure 2. In comparative ROC analysis, the TG/HDL-C ratio showed greater discrimination than triglycerides alone (difference in AUC: 0.031, DeLong *p* < 0.001), whereas its AUC did not differ significantly from that of low HDL-C (difference in AUC: −0.015, *p* = 0.325). Detailed pairwise comparisons are presented in Supplementary Table S2.

### 3.5. Multivariable Logistic Regression Analyses

In multivariable logistic regression analysis, the TG/HDL-C ratio remained associated with poor glycemic control after adjustment for age, sex, LDL-C, total cholesterol, insulin use, statin use, and fenofibrate use. Each one-unit increase in the TG/HDL-C ratio was associated with 20.1% higher odds of HbA1c ≥ 7% (OR: 1.201, 95% CI: 1.125–1.282, *p* < 0.001). Insulin use and statin use were also associated with poor glycemic control, whereas age, sex, and LDL-C were not. The model showed acceptable calibration (Hosmer–Lemeshow *p* = 0.573), with a Nagelkerke R^2^ of 0.125. No relevant multicollinearity was detected, with VIF values ranging from 1.009 to 2.007. Internal validation using 1000 bootstrap resamples supported the stability of the association between the TG/HDL-C ratio and poor glycemic control (B = 0.183, bootstrap *p* = 0.001, BCa 95% CI: 0.121–0.261). The results of the continuous TG/HDL-Cmodel are presented in Table 5.

A tertile-based logistic regression model was also constructed using the lowest TG/HDL-Ctertile as the reference category. Compared with patients in the lowest tertile, those in the intermediate tertile had higher odds of poor glycemic control (OR: 1.562, 95% CI: 1.133–2.155, *p* = 0.007), as did those in the highest tertile (OR: 2.733, 95% CI: 1.925–3.881, *p* < 0.001). The tertile-based model also showed acceptable calibration (Hosmer–Lemeshow *p* = 0.299). These results are shown in Table 6.

The model fit statistics for the tertile-based model are presented in Table 7.

### 3.6. Sensitivity Analysis

To address the potential confounding effect of fenofibrate therapy on the TG/HDL-C ratio, a sensitivity analysis was performed after excluding patients receiving fenofibrate therapy; 908 patients remained. In this subgroup, the TG/HDL-C ratio remained significantly associated with poor glycemic control in the continuous model (OR: 1.179, 95% CI: 1.098–1.266, *p* < 0.001). In the tertile-based model, the intermediate and highest tertiles remained significantly associated with HbA1c ≥ 7% compared with the lowest tertile (OR: 1.645, 95% CI: 1.186–2.281, *p* = 0.003; and OR: 2.438, 95% CI: 1.703–3.490, *p* < 0.001, respectively). These findings are presented in Supplementary Table S1.

In both regression models, all VIF values were below 3.0, indicating no relevant multicollinearity among the predictors. The completed STROBE checklist is provided in Table S3.

## 4. Discussion

In this real-world cohort of 1001 patients with T2DM, the TG/HDL-C ratio was significantly associated with glycemic control and adverse metabolic characteristics. Patients with HbA1c ≥ 7% had higher TG/HDL-C ratios than those with HbA1c < 7%, and HbA1c levels increased progressively across TG/HDL-C tertiles. In multivariable logistic regression analysis, the TG/HDL-C ratio remained associated with poor glycemic control after adjustment for the covariates available in the present dataset, and patients in the highest tertile had markedly higher odds of HbA1c ≥ 7% than those in the lowest tertile. These findings suggest that the TG/HDL-C ratio reflects an unfavorable metabolic phenotype in patients with T2DM.

The association between the TG/HDL-C ratio and poor glycemic control is biologically plausible. Hypertriglyceridemia and low HDL-C are characteristic components of diabetic dyslipidemia and are closely related to insulin resistance, hepatic overproduction of very-low-density lipoprotein particles, impaired lipoprotein lipase activity, and increased exchange of triglycerides and cholesterol esters between lipoproteins [7]. The TG/HDL-C ratio integrates these two lipid abnormalities into a single index and has been proposed as a surrogate marker of insulin resistance and cardiometabolic risk [13,14,15]. In the present study, the ratio showed significant positive correlations with HbA1c and fasting plasma glucose, supporting its relationship with the degree of glycemic dysregulation. Beyond insulin resistance and altered lipid metabolism, oxidative stress and endothelial dysfunction may represent additional biological pathways linking an adverse metabolic profile with impaired glucose homeostasis. An unfavorable redox environment can reduce nitric oxide bioavailability and disrupt physiological nitric oxide-dependent endothelial signaling, whereas excessive interaction between nitric oxide and reactive oxygen species may promote reactive nitrogen species formation, nitrosative stress, and cellular dysfunction. These mechanisms may contribute to vascular and metabolic disturbances accompanying dyslipidemia; however, they should be regarded as biologically plausible pathways rather than as mechanisms directly demonstrated by the present observational study [32].

Chronic low-grade inflammation may provide an additional biological link between metabolic dysfunction and impaired glycemic regulation. Metabolic abnormalities and inflammatory activation are unlikely to represent independent processes; rather, inflammatory cytokine networks and intracellular signaling pathways may interact with insulin resistance and disturbances in lipid metabolism, potentially amplifying metabolic dysfunction. Such interconnected signaling may help explain the coexistence of adverse lipid profiles and impaired glucose homeostasis. However, inflammatory mediators were not measured in the present study, and this mechanism therefore remains a biologically plausible interpretation rather than a directly demonstrated pathway [33].

Although statistically significant, the discriminatory performance of the TG/HDL-C ratio was limited (AUC 0.601), indicating that statistical significance should not be interpreted as strong clinical classification ability. The corresponding sensitivity of 58.1% and specificity of 56.6% further support its limited value as a standalone clinical discriminator. These observations are consistent with prior reports in which the TG/HDL-C ratio showed weak or inconsistent associations with glycemic control, and in which glucose-containing indices such as the triglyceride–glucose index demonstrated stronger discriminatory performance [23,24,25,26,27]. Therefore, the TG/HDL-C ratio should not be interpreted as a standalone diagnostic test for poor glycemic control, but rather as a simple, inexpensive, and readily available metabolic marker that provides additional context regarding cardiometabolic risk and glycemic status. The practical advantage of the TG/HDL-C ratio over its individual lipid components also appears limited. In comparative ROC analyses, the TG/HDL-C ratio showed greater discrimination than triglycerides alone, but its AUC did not differ significantly from that of HDL-C. Therefore, the ratio should not be considered clearly superior to conventional lipid parameters for identifying poor glycemic control. Its potential value may instead lie in providing a simple composite representation of the triglyceride–HDL-C metabolic pattern using routinely available laboratory measurements.

Importantly, statistical association should be distinguished from clinical discrimination. Although the TG/HDL-C ratio remained associated with poor glycemic control in multivariable analyses, the magnitude of its correlations with HbA1c and fasting plasma glucose was small, and its ROC performance was limited (AUC 0.601). The corresponding sensitivity of 58.1% and specificity of 56.6% further indicate that the ratio lacks sufficient standalone discriminatory ability for clinical classification of glycemic control. Accordingly, the TG/HDL-C ratio should be interpreted as an adjunctive marker of an adverse metabolic phenotype rather than as a diagnostic or predictive test.

The tertile-based analysis further supports the clinical relevance of the TG/HDL-C ratio. HbA1c and fasting plasma glucose increased progressively from the lowest to the highest tertile, and the odds of poor glycemic control showed a graded increase across tertiles after multivariable adjustment. This graded pattern supports the consistency of the observed association across increasing TG/HDL-C levels.

Because lipid-lowering therapies may influence triglyceride and HDL-C levels, treatment-related confounding is an important consideration. In the present study, statin and fenofibrate use were included as covariates in the primary multivariable models, and a sensitivity analysis was performed after excluding patients receiving fenofibrate therapy, because fenofibrate may directly reduce triglyceride levels and modify the TG/HDL-C ratio. However, because the exact timing and duration of insulin, statin, and fenofibrate therapy relative to laboratory assessment were unavailable, treatment-related temporal effects could not be fully evaluated. The association between the ratio and poor glycemic control remained significant after exclusion of fenofibrate users in both the continuous and tertile-based models, suggesting that the observed association was not solely driven by fenofibrate therapy. Because categorizing a continuous variable may reduce information and statistical efficiency, the tertile-based findings were interpreted as complementary to the primary continuous-variable analysis rather than as the principal estimate of association.

The findings should be interpreted in light of the study design. Given the retrospective observational design, the findings demonstrate association rather than causation. A higher TG/HDL-C ratio should therefore not be interpreted as causing poorer glycemic control. Although the TG/HDL-C ratio remained associated with poor glycemic control, it cannot be concluded that a higher ratio causes higher HbA1c levels. The relationship may reflect shared underlying mechanisms such as insulin resistance, obesity-related metabolic dysfunction, dietary patterns, physical inactivity, or unmeasured inflammatory processes. The strong association between insulin use and poor glycemic control should therefore not be interpreted as indicating an adverse effect of insulin therapy. Insulin use may instead represent confounding by indication, because patients with longer-standing or more severe diabetes and those with previously inadequate glycemic control are more likely to receive insulin. Furthermore, because the exact timing of insulin initiation relative to HbA1c measurement was unavailable, the temporal direction of this association could not be established.

This study has several strengths. First, it included a large real-world cohort of patients with T2DM, improving the clinical relevance of the findings. Second, the TG/HDL-C ratio was evaluated using multiple complementary approaches, including group comparisons, correlation analysis, ROC analysis, tertile-based comparisons, and multivariable logistic regression. Third, treatment-related confounding was addressed by including lipid-lowering therapies in the regression models and by performing a sensitivity analysis excluding fenofibrate users. Fourth, multicollinearity was assessed and no relevant multicollinearity was detected. Although the multivariable model showed acceptable calibration, its Nagelkerke R^2^ was 0.125, indicating that the included variables explained only a limited proportion of variation in glycemic-control status. This finding further supports the likely contribution of additional clinical, anthropometric, treatment-related, and lifestyle factors not captured in the present dataset.

Several limitations should also be acknowledged. The retrospective and single-country design may limit generalizability. Data on diabetes duration, body mass index, and waist circumference were not available for the present analysis, while several other potentially relevant factors, including smoking status, renal function, lifestyle characteristics, and detailed glucose-lowering treatment, could not be comprehensively incorporated into the primary multivariable models. Because these factors may influence both the TG/HDL-C ratio and glycemic control, residual confounding cannot be excluded. Consequently, the adjusted association observed in this study should not be interpreted as demonstrating an association independent of all relevant metabolic, clinical, and treatment-related determinants. Detailed information on the initiation and duration of insulin, statin, and fenofibrate therapy relative to the corresponding laboratory measurements was unavailable, limiting assessment of temporal treatment-related effects. The lack of class-specific glucose-lowering treatment data may have contributed to residual treatment-related confounding, because individual therapies may differentially affect HbA1c, body weight, insulin sensitivity, and lipid parameters. Detailed information on the type, dose, and intensity of lipid-lowering therapy was not available. In addition, no patients were receiving gemfibrozil or PCSK9 inhibitor therapy and only one patient was receiving ezetimibe, limiting the ability to evaluate the potential effects of other lipid-modifying therapies. Although internal validation was performed using bootstrap resampling, external validation in an independent population was not available; therefore, the generalizability of the observed associations and model performance requires confirmation in other clinical settings. These unmeasured variables may confound the association between the TG/HDL-C ratio and glycemic control, and the absence of a direct insulin-resistance measure (such as fasting insulin or the homeostatic model assessment of insulin resistance [HOMA-IR]) precluded formal evaluation of the ratio as an insulin-resistance surrogate in this cohort. The study used a single time-point assessment of laboratory values, so longitudinal changes in the TG/HDL-C ratio and HbA1c could not be evaluated. In addition, although the ratio was significantly associated with HbA1c ≥ 7%, its ROC performance was modest, limiting its role as an isolated predictive tool. Finally, because this was a secondary analysis of an existing dataset, the findings should be considered hypothesis-generating and validated in prospective cohorts.

Future prospective studies should validate the observed association between the TG/HDL-C ratio and glycemic control in independent and more diverse populations, with more comprehensive adjustment for anthropometric, lifestyle, renal, and treatment-related factors. Further research should also evaluate whether serial changes in the TG/HDL-C ratio provide incremental information beyond established glycemic and metabolic markers. Given its limited standalone discriminatory performance in the present study, the TG/HDL-C ratio should primarily be investigated as an adjunctive metabolic marker rather than as a diagnostic or predictive tool.

## 5. Conclusions

The TG/HDL-C ratio was associated with poor glycemic control in patients with T2DM and showed a graded relationship with HbA1c across tertiles. However, its standalone discriminatory performance was limited. These findings should also be interpreted in light of residual treatment-related confounding, particularly the absence of class-specific glucose-lowering therapy data. Therefore, the TG/HDL-C ratio should not be considered a diagnostic or predictive tool for poor glycemic control; rather, it may represent a simple adjunctive metabolic marker associated with an adverse glycemic and atherogenic profile.

## Figures and Tables

**Table 1:** Baseline Characteristics of Patients.

Variable	Value
Age (years)	60.9 ± 11.4
Female (n, %)	572 (57.1%)
Male (n, %)	429 (42.9%)
HbA1c (%)	7.2 (6.3–8.3)
Fasting Plasma Glucose (mg/dL)	134 (110–169)
HDL (mg/dL)	48 (40–56)
LDL (mg/dL)	110.7 ± 42.8
Triglycerides (mg/dL)	150 (107–211)
TG/HDL-C ratio	3.16 (2.11–4.75)

Note: Continuous variables are presented as mean ± standard deviation or median (Q1–Q3), according to their distribution; categorical variables are presented as n (%). Statistical significance was set at *p* < 0.05. HbA1c, glycated hemoglobin; HDL, high-density lipoprotein cholesterol; LDL, low-density lipoprotein cholesterol; TG, triglycerides.

**Table 2:** Comparison of clinical and metabolic parameters according to glycemic control status.

Variable	HbA1c < 7 n = 433	HbA1c ≥ 7 n = 568	*p*-Value
Age, years	61.0 (53.5–68.0)	62.0 (54.0–69.0)	0.302
Fasting plasma glucose, mg/dL	114.0 (98.0–134.0)	158.0 (124.0–197.0)	**<0.001**
HDL-C, mg/dL	52.0 (42.5–60.0)	46.0 (39.0–53.0)	**<0.001**
LDL-C, mg/dL	117.0 (91.0–143.0)	105.0 (79.0–139.8)	**<0.001**
Total cholesterol, mg/dL	198.0 (172.0–226.0)	193.0 (157.0–230.0)	0.034
Triglycerides, mg/dL	145.0 (96.0–205.0)	158.0 (119.0–217.0)	**<0.001**
**TG/HDL-C ratio**	**2.74 (1.87–3.82)**	**3.35 (2.35–5.00)**	**<0.001**
Female sex, n (%)	255 (58.9%)	317 (55.8%)	0.329
Insulin use, n (%)	41 (9.5%)	143 (25.2%)	**<0.001**
Neuropathy drug use, n (%)	42 (9.7%)	72 (12.7%)	0.142
Fenofibrate use, n (%)	38 (8.8%)	55 (9.7%)	0.624
Statin use, n (%)	117 (27.0%)	182 (32.0%)	0.08

Note: Continuous variables are presented as median (interquartile range [IQR]). Categorical variables are presented as n (%). Statistical significance was set at *p* < 0.001 or *p* < 0.05. HbA1c, glycated hemoglobin; HDL-C, high-density lipoprotein cholesterol; LDL-C, low-density lipoprotein cholesterol; TG, triglycerides. Bold values indicate statistical significance (*p* < 0.05).

**Table 3:** Correlation analysis between TG/HDL-C ratio and clinical-metabolic parameters.

Variable	Spearman’s Rho	95% CI	*p*-Value
Age, years	−0.065	−0.128 to −0.001	0.039
HbA1c, %	0.172	0.109 to 0.233	**<0.001**
Fasting plasma glucose, mg/dL	0.168	0.106 to 0.230	**<0.001**
LDL-C, mg/dL	−0.008	−0.072 to 0.055	0.789
Total cholesterol, mg/dL	0.208	0.147 to 0.269	**<0.001**

Note: Correlations were assessed using Spearman’s rank correlation analysis. CI, confidence interval; HDL-C, high-density lipoprotein cholesterol; LDL-C, low-density lipoprotein cholesterol; TG/HDL-C, triglyceride-to-HDL cholesterol ratio. Bold values indicate statistical significance (*p* < 0.05).

**Table 4:** Clinical and metabolic characteristics according to TG/HDL-C ratio tertiles.

Variable	Tertile 1 Low TG/HDL-C n = 333	Tertile 2 Intermediate TG/HDL-C n = 335	Tertile 3 High TG/HDL-C n = 333	*p*-Value
TG/HDL-C ratio	1.79 (1.35–2.11)	3.16 (2.72–3.48)	5.76 (4.75–7.70)	**<0.001**
Age, years	62.0 (54.0–69.0)	62.0 (54.0–69.0)	60.0 (52.0–68.0)	0.087
HbA1c, %	6.90 (6.20–7.90)	7.20 (6.30–8.20)	7.60 (6.50–8.70)	**<0.001**
Fasting plasma glucose, mg/dL	124.0 (105.0–157.0)	138.0 (114.0–169.0)	146.0 (113.5–192.0)	**<0.001**
HDL-C, mg/dL	55.0 (50.0–63.0)	48.0 (44.0–56.0)	39.0 (34.0–46.0)	**<0.001**
LDL-C, mg/dL	107.0 (83.0–136.5)	123.0 (91.0–146.0)	107.0 (75.5–137.0)	**<0.001**
Total cholesterol, mg/dL	182.0 (152.5–220.0)	200.0 (171.0–229.0)	204.0 (170.0–239.0)	**<0.001**
Triglycerides, mg/dL	92.0 (76.0–116.5)	150.0 (129.0–175.0)	234.0 (199.0–296.5)	**<0.001**
Female sex, n (%)	210 (63.1%)	191 (57.0%)	171 (51.4%)	**0.009**
Insulin use, n (%)	55 (16.5%)	68 (20.3%)	61 (18.3%)	0.451
Statin use, n (%)	111 (33.3%)	115 (34.3%)	73 (21.9%)	**0.001**
Fenofibrate use, n (%)	4 (1.2%)	19 (5.7%)	70 (21.0%)	**<0.001**

Note: Continuous variables are presented as median (interquartile range), and categorical variables are presented as number and percentage. *p*-values were calculated using the Kruskal–Wallis test for continuous variables and the chi-square test for categorical variables. HbA1c, glycated hemoglobin; HDL-C, high-density lipoprotein cholesterol; LDL-C, low-density lipoprotein cholesterol; TG/HDL-C, triglyceride-to-high-density lipoprotein cholesterol ratio. For HbA1c, post-hoc pairwise comparisons were performed using Mann–Whitney U tests with Bonferroni correction for three comparisons (adjusted significance threshold, *p* < 0.0167). HbA1c differed significantly between tertiles 1 and 3 (*p* < 0.001) and between tertiles 2 and 3 (*p* = 0.001), whereas the difference between tertiles 1 and 2 was not statistically significant (*p* = 0.051). Bold values indicate statistical significance (*p* < 0.05).

**Table 5:** Logistic regression analysis for predictors of poor glycemic control.

Variable	OR	95% CI	*p*-Value
TG/HDL-C ratio	1.201	1.125–1.282	**<0.001**
Age, years	1.003	0.991–1.014	0.660
Female sex	1.005	0.767–1.318	0.969
LDL-C, mg/dL	0.999	0.994–1.004	0.666
Total cholesterol, mg/dL	0.995	0.991–0.999	0.027
Insulin use	3.311	2.252–4.854	**<0.001**
Statin use	1.515	1.130–2.033	**0.006**
Fenofibrate use	0.625	0.372–1.050	0.076

Note: Logistic regression analysis was performed with poor glycemic control, defined as HbA1c ≥ 7%, as the dependent variable. ORs for insulin use, statin use, and fenofibrate use were presented after reversing the original SPSS reference coding to provide clinically intuitive estimates. CI, confidence interval; HDL-C, high-density lipoprotein cholesterol; LDL-C, low-density lipoprotein cholesterol; OR, odds ratio; TG/HDL-C, triglyceride-to-HDL cholesterol ratio. Bold values indicate statistical significance (*p* < 0.05).

**Table 6:** Logistic regression analysis of TG/HDL-C ratio tertiles for poor glycemic control.

Variable	OR	95% CI	*p*-Value
TG/HDL-Ctertile 1, low	Reference	—	—
TG/HDL-Ctertile 2, intermediate	1.562	1.133–2.155	**0.007**
TG/HDL-Ctertile 3, high	2.733	1.925–3.881	**<0.001**
Age, years	1.003	0.991–1.014	0.646
Female sex	1.013	0.773–1.330	0.923
LDL-C, mg/dL	0.995	0.991–0.999	0.018
Total cholesterol, mg/dL	0.997	0.994–1.001	0.184
Insulin use	3.448	2.347–5.051	**<0.001**
Statin use	1.495	1.112–2.004	**0.008**
Fenofibrate use	0.700	0.425–1.155	0.162

Note: Logistic regression analysis was performed with poor glycemic control, defined as HbA1c ≥ 7%, as the dependent variable. TG/HDL-Ctertile 1 was used as the reference category. ORs for insulin use, statin use, and fenofibrate use were presented after reversing the original SPSS reference coding to provide clinically intuitive estimates. CI, confidence interval; HDL-C, high-density lipoprotein cholesterol; LDL-C, low-density lipoprotein cholesterol; OR, odds ratio; TG/HDL-C, triglyceride-to-HDL cholesterol ratio. Bold values indicate statistical significance (*p* < 0.05).

**Table 7:** Model fit statistics for the tertile-based model.

Model Parameter	Value
Omnibus model *p*-value	<0.001
Nagelkerke R^2^	0.121
Hosmer–Lemeshow *p*-value	0.299
Overall classification accuracy	62.2%

## Data Availability

The datasets generated and/or analyzed during the current study are not publicly available due to institutional and ethical restrictions but can be made available from the corresponding author upon reasonable request.

## Supplementary Materials

The following supporting information can be downloaded at: https://www.mdpi.com/article/10.3390/biomedicines14091995/s1. Table S1: Sensitivity analysis excluding patients receiving fenofibrate therapy; Table S2: Comparative ROC analysis of the TG/HDL-C ratio and its lipid components for discriminating poor glycemic control; Table S3: STROBE checklist.
