# Supplementary material for: Association Between the Triglyceride-to-HDL Cholesterol Ratio and Glycemic Control in Patients with Type 2 Diabetes Mellitus: A Real-World Retrospective Study
Source: Biomedicines. 2026 Sep 4;14(9):1995. doi: 10.3390/biomedicines14091995 (PMC13604466; doi:10.3390/biomedicines14091995)
Supplement: Supplementary file 1 [file biomedicines-14-01995-s001.zip › Table S3-STROBE Checklist.pdf]

STROBE Statement—Checklist of Items That Should Be Included in Reports of Observational Studies

Completed for: Association Between the Triglyceride-to-HDL Cholesterol Ratio and Glycemic Control in Patients with Type 2 Diabetes Mellitus: A Real-World Retrospective Study

Section / Item	No.	STROBE recommendation	Page(s)	Relevant text / location in manuscript
Title and abstract	1	(a) Indicate the study's design with a commonly used term in the title or the abstract	1	Title identifies the work as a "Real-World Retrospective Study"; the Abstract also states that this was a retrospective observational study.
Title and abstract	1	(b) Provide in the abstract an informative and balanced summary of what was done and what was found	1–2	The Abstract summarizes the background, methods, study population, adjusted association, sensitivity analysis, ROC performance, and conservative conclusion.
**Introduction**				
Background/rationale	2	Explain the scientific background and rationale for the investigation being reported	2–3	Introduction describes the global burden of T2DM, diabetic dyslipidemia, insulin resistance, prior TG/HDL-C evidence, and the need to clarify its association with glycemic control.
Objectives	3	State specific objectives, including any prespecified hypotheses	1, 3	Objective: to evaluate the association between the TG/HDL-C ratio and poor glycemic control in patients with T2DM; the secondary-analysis rationale and distinct analytical framework are also stated.
**Methods**				
Study design	4	Present key elements of study design early in the paper	3	Section 2.1 identifies the study as a retrospective observational secondary analysis of anonymized electronic medical-record data.
Setting	5	Describe the setting, locations, and relevant dates, including periods of recruitment, exposure, follow-up, and data collection	3	Setting: Internal Medicine outpatient clinics of Kestel State Hospital and Bursa Yüksek İhtisas Training and Research Hospital, Bursa, Türkiye; study period March 2024–March 2025.
Participants	6	(a) Cohort study—Give the eligibility criteria, and the sources and methods of selection of participants. Describe methods of follow-up<br>Case-control study—Give the eligibility criteria, and the sources and methods of case ascertainment and control selection. Give the rationale for the choice of cases and controls<br>Cross-sectional study—Give the eligibility criteria, and the sources and methods of selection of participants	3–4	Eligibility criteria are provided: adults aged 18–90 years with confirmed T2DM and required laboratory data. Exclusions included type 1 diabetes, gestational diabetes, missing essential laboratory data, and severe comorbid conditions that could substantially interfere with lipid/metabolic measurements.
Participants	6	(b) Cohort study—For matched studies, give matching criteria and number of exposed and unexposed<br>Case-control study—For matched studies, give matching criteria and the number of controls per case	N/A	No formal matching was performed. The study was not a matched cohort or case-control study.
Variables	7	Clearly define all outcomes, exposures, predictors, potential confounders, and effect modifiers. Give diagnostic criteria, if applicable	4–5	HbA1c ≥7% was defined as poor glycemic control; HbA1c <7% as better control. TG/HDL-C calculation, medication exposures, tertiles, covariates, and regression outcome are defined.
Data sources/ measurement	8*	For each variable of interest, give sources of data and details of methods of assessment (measurement). Describe comparability of assessment methods if there is more than one group	4	Demographic, clinical, laboratory, and treatment data were retrieved from electronic hospital records. Fasting laboratory collection, contemporaneous HbA1c assessment, medication-exposure ascertainment, and TG/HDL-C calculation are described.
Bias	9	Describe any efforts to address potential sources of bias	4–5, 13–14	Potential bias/confounding was addressed by a priori covariate adjustment, fenofibrate sensitivity analysis, multicollinearity assessment, bootstrap internal validation, and explicit discussion of residual confounding and treatment-timing limitations.
Study size	10	Explain how the study size was arrived at	4	No a priori sample-size calculation was performed; all eligible records in the predefined study period were included. Final sample size reflected the available eligible source population.
Quantitative variables	11	Explain how quantitative variables were handled in the analyses. If applicable, describe which groupings were chosen and why	4–5	TG/HDL-C was analyzed primarily as a continuous variable and secondarily in tertiles to evaluate graded patterns while preserving the continuous analysis as primary. HbA1c was dichotomized at 7% according to ADA guidance.
Statistical methods	12	(a) Describe all statistical methods, including those used to control for confounding	5	Statistical methods include normality testing, descriptive statistics, Mann–Whitney U, chi-square, Kruskal–Wallis with Bonferroni-adjusted pairwise tests, effect sizes, Spearman correlation, ROC/Youden analysis, multivariable logistic regression, VIF/tolerance, and bootstrap validation.
Statistical methods	12	(b) Describe any methods used to examine subgroups and interactions	4–5, 7–10	Secondary analyses included TG/HDL-C tertile analyses and a tertile-based logistic regression model. No interaction analysis was reported.
Statistical methods	12	(c) Explain how missing data were addressed	3–4	Availability of essential laboratory data was an eligibility requirement; records with missing essential laboratory data were excluded. No additional missing-data imputation procedure was reported.
Statistical methods	12	(d) Cohort study—If applicable, explain how loss to follow-up was addressed<br>Case-control study—If applicable, explain how matching of cases and controls was addressed<br>Cross-sectional study—If applicable, describe analytical methods taking account of sampling strategy	N/A	No longitudinal follow-up, matched design, or complex sampling strategy was used in this retrospective secondary analysis.
Statistical methods	12	(e) Describe any sensitivity analyses	5, 11	Sensitivity analysis excluded patients receiving fenofibrate and repeated both continuous and tertile-based regression models.
**Results**				
Participants	13*	(a) Report numbers of individuals at each stage of study—eg numbers potentially eligible, examined for eligibility, confirmed eligible, included in the study, completing	5–6	A total of 1,001 patients were included in the final analysis. The manuscript states that all eligible records from the predefined period were included.

Section / Item	No.	STROBE recommendation	Page(s)	Relevant text / location in manuscript
		follow-up, and analysed		
Participants	13*	(b) Give reasons for non-participation at each stage	3–4	Reasons for exclusion are specified in the eligibility criteria (type 1 diabetes, gestational diabetes, missing essential laboratory data, and severe comorbid conditions affecting lipid/metabolic measurements); stage-specific exclusion counts are not separately reported.
Participants	13*	(c) Consider use of a flow diagram	N/A	No participant-flow diagram is included; the manuscript reports the final eligible sample and eligibility/exclusion criteria.
Descriptive data	14*	(a) Give characteristics of study participants (eg demographic, clinical, social) and information on exposures and potential confounders	5–8	Participant age, sex, HbA1c, fasting glucose, lipid parameters, TG/HDL-C ratio, and medication exposures are reported in Table 1, Table 2, and the tertile analyses.
Descriptive data	14*	(b) Indicate number of participants with missing data for each variable of interest	3–4	Included participants were required to have the essential laboratory variables available; records missing essential laboratory data were excluded. Variable-specific missing counts among included participants are not reported.
Descriptive data	14*	(c) Cohort study—Summarise follow-up time (eg, average and total amount)	N/A	No follow-up time applies; this was a retrospective analysis of clinical assessments rather than a longitudinal cohort follow-up study.
Outcome data	15*	Cohort study—Report numbers of outcome events or summary measures over time	N/A	Not a longitudinal cohort outcome analysis.
Outcome data	15*	Case-control study—Report numbers in each exposure category, or summary measures of exposure	N/A	Not a case-control study.
Outcome data	15*	Cross-sectional study—Report numbers of outcome events or summary measures	6–11	Poor glycemic control was present in 568/1,001 (56.7%) patients; group summaries, tertile distributions, ROC results, regression estimates, and sensitivity-analysis results are reported.
Main results	16	(a) Give unadjusted estimates and, if applicable, confounder-adjusted estimates and their precision (eg, 95% confidence interval). Make clear which confounders were adjusted for and why they were included	6–11	Unadjusted group comparisons and correlations are reported; adjusted logistic-regression ORs with 95% CIs are presented for the continuous and tertile models. Covariates were selected a priori for clinical relevance and confounding potential.
Main results	16	(b) Report category boundaries when continuous variables were categorized	4, 7–10	TG/HDL-C was categorized into low, intermediate, and high sample tertiles for secondary analyses; group sizes and tertile-specific distributions are reported. Exact numerical tertile cut-points are not explicitly stated in the manuscript.
Main results	16	(c) If relevant, consider translating estimates of relative risk into absolute risk for a meaningful time period	N/A	Absolute-risk translation over time is not applicable because this analysis did not estimate longitudinal risk over a defined follow-up period.
Other analyses	17	Report other analyses done—eg analyses of subgroups and interactions, and sensitivity analyses	7–11	Additional analyses included post-hoc pairwise tertile comparisons with effect sizes, comparative ROC analysis, tertile-based logistic regression, model diagnostics/bootstrap validation, and fenofibrate-exclusion sensitivity analysis.
**Discussion**				
Key results	18	Summarise key results with reference to study objectives	11–14	Discussion summarizes the main findings: higher TG/HDL-C was associated with poorer glycemic control, the association was graded across tertiles and persisted after adjustment/sensitivity analysis, while standalone discrimination was limited.
Limitations	19	Discuss limitations of the study, taking into account sources of potential bias or imprecision. Discuss both direction and magnitude of any potential bias	13–14	Limitations include retrospective design, residual confounding, unavailable BMI/diabetes duration/waist circumference, smoking/renal/lifestyle factors, class-specific glucose-lowering therapy, medication timing/duration, sparse other lipid therapies, lack of direct insulin-resistance measures, single time point, and lack of external validation.
Interpretation	20	Give a cautious overall interpretation of results considering objectives, limitations, multiplicity of analyses, results from similar studies, and other relevant evidence	11–14	Interpretation is explicitly cautious: association is distinguished from causation and from clinical discrimination; AUC 0.601, sensitivity 58.1%, and specificity 56.6% are interpreted as limited standalone performance.
Generalisability	21	Discuss the generalisability (external validity) of the study results	13–14	The manuscript notes that the retrospective single-country design may limit generalizability and calls for prospective validation in independent, more diverse populations.
**Other information**				
Funding	22	Give the source of funding and the role of the funders for the present study and, if applicable, for the original study on which the present article is based	14	Funding statement: “This research received no external funding.” No funder role therefore applies to the present study.

N/A = not applicable. Page numbers refer to the current 17-page Biomedicines manuscript version supplied for checklist completion.
