# Supplementary material for: Association Between the Triglyceride-to-HDL Cholesterol Ratio and Glycemic Control in Patients with Type 2 Diabetes Mellitus: A Real-World Retrospective Study
Source: Biomedicines. 2026 Sep 4;14(9):1995. doi: 10.3390/biomedicines14091995 (PMC13604466; doi:10.3390/biomedicines14091995)
Supplement: Supplementary file 1 [file biomedicines-14-01995-s001.zip › Tables S1 and S2.pdf]

Supplementary Table S1. Sensitivity analysis excluding patients receiving fenofibrate therapy

Panel A. Continuous TG/HDL-C Ratio model

**Variable**	**OR**	**95% CI**	**P-value**
TG/HDL-C ratio	1.179	1.098–1.266	**<0.001**
Age, years	1.003	0.991–1.016	0.577
Female sex	1.006	0.758–1.336	0.968
LDL-C, mg/dL	1.001	0.995–1.007	0.773
Total cholesterol, mg/dL	0.994	0.988–0.999	**0.020**
Insulin use	3.205	2.146–4.785	**<0.001**
Statin use	1.464	1.083–1.980	**0.013**

Panel B. TG/HDL tertile-based model

**Variable**	**OR**	**95% CI**	**P-value**
TG/HDL-C ratio tertile 1, low	1.00	Reference	—
TG/HDL-C ratio tertile 2, intermediate	1.645	1.186–2.281	**0.003**
TG/HDL-C ratio tertile 3, high	2.438	1.703–3.490	**<0.001**
Age, years	1.003	0.991–1.015	0.673
Female sex	1.035	0.778–1.377	0.812
LDL-C, mg/dL	0.998	0.993–1.004	0.548
Total cholesterol, mg/dL	0.995	0.990–1.000	0.051
Insulin use	3.268	2.183–4.902	**<0.001**
Statin use	1.427	1.055–1.931	**0.021**

Note: Sensitivity analyses were performed after excluding patients receiving fenofibrate therapy. Poor glycemic control, defined as HbA1c ≥7%, was used as the dependent variable. Panel A included TG/HDL ratio as a continuous variable, whereas Panel B included TG/HDL tertiles, with the lowest tertile used as the reference category. Odds ratios for insulin use and statin use were presented after reversing the original SPSS reference coding to provide clinically intuitive estimates. CI, confidence interval; HDL-C, high-density lipoprotein cholesterol; LDL-C, low-density lipoprotein cholesterol; OR, odds ratio; TG/HDL-C ratio, triglyceride-to-HDL cholesterol ratio. After excluding 93 fenofibrate users, 908 patients remained.

Supplementary Table S2. Comparative ROC analysis of TG/HDL-C ratio and its lipid components for predicting poor glycemic control

Panel A. ROC performance of lipid markers

Marker	AUC	95% CI	P-value
TG/HDL-C ratio	0.601	0.566–0.636	**<0.001**
Triglycerides	0.569	0.534–0.605	**<0.001**
Low HDL-C	0.616	0.581–0.651	**<0.001**

Panel B. Pairwise comparison of AUC values using DeLong test

Comparison	Difference in AUC	DeLong P-value
TG/HDL-C ratio vs triglycerides	+0.031	**<0.001**
TG/HDL-C ratio vs low HDL-C	-0.015	0.325
Triglycerides vs low HDL-C	-0.047	0.036

Note: *Poor glycemic control was defined as HbA1c ≥7%. Because lower HDL-C values indicate higher metabolic risk, HDL-C was analyzed in the inverse direction for ROC analysis. Pairwise AUC comparisons were performed using the paired DeLong test. AUC, area under the curve; CI, confidence interval; HDL-C, high-density lipoprotein cholesterol; TG/HDL-C ratio, triglyceride-to-HDL cholesterol ratio.*
